# IL-9 Induces CCL11 Expression via STAT3 Signalling in Human Airway Smooth Muscle Cells

**DOI:** 10.1371/journal.pone.0009178

**Published:** 2010-02-12

**Authors:** Akira Yamasaki, Ali Saleh, Latifa Koussih, Shigeo Muro, Andrew J. Halayko, Abdelilah S. Gounni

**Affiliations:** 1 Department of Immunology, University of Manitoba, Winnipeg, Manitoba, Canada; 2 Department of Physiology, University of Manitoba, Winnipeg, Manitoba, Canada; 3 Respiratory Section, University of Manitoba, Winnipeg, Manitoba, Canada; 4 Department of Respiratory Medicine, Kyoto University Hospital, Sakyo-ku, Kyoto, Japan; University of Alabama-Birmingham, United States of America

## Abstract

**Background:**

Previous findings support the concept that IL-9 may play a significant role in mediating both pro-inflammatory and changes in airway responsiveness that characterizes the atopic asthmatic state. We previously demonstrated that human airway smooth muscle (ASM) cells express a functional IL-9R that mediate CCL11 expression. However, the signaling pathway governing this effect is not well understood.

**Methodology/Principal Findings:**

In this study, we showed that IL-9 mediated CCL11 expression in ASM cells does not rely on STAT6 or STAT5 but on STAT3 pathway. IL-9 induced rapid STAT3 activation in primary ASM cells that was not observed in case of STAT6 or STAT5. STAT3 binding to CCL11 promoter was also observed *in vivo* upon IL-9 stimulation of ASM cells. Disruption of STAT3 activity with SH2 domain binding inhibitory peptide results in significant reduction of IL-9 mediated CCL11 promoter activity. DN STAT3β over-expression in ASM cells, but not Ser 727 STAT3 or STAT6 DN, abolishes IL-9 mediated CCL11 promoter activity. Finally, STAT3 but not STAT6 silenced ASM cells showed significant reduction in IL-9 mediated CCL11 promoter activity and mRNA expression.

**Conclusion/Significance:**

Taken together, our results indicate that IL-9 mediated CCL11 via STAT3 signalling pathway may play a crucial role in airway inflammatory responses.

## Introduction

Airway smooth muscle (ASM) cells are key structural cells involved in the pathogenesis of many airway diseases by contributing to inflammation and airway hyperresponsiveness[1]. In addition to their proliferative and contractile properties, studies suggest that ASM cells can contribute directly to the pathogenesis of asthma by expressing cell adhesion and costimulatory molecules and by secreting multiple proinflammatory cytokines and chemokines that may perpetuate airway inflammation and the development of airway remodeling *in vivo* [2].

IL-9 is a T cell derived cytokine with pleiotropic activities on various cell types[3]. The expression of IL-9 is detectable mainly in activated CD4+T cells. It has been shown that IL-9 can promote the proliferation of activated and transformed T cells, the production of immunoglobulins by B cells, the proliferation and differentiation of mast cells, and erythroid progenitors[4]. A number of observations have suggested that this cytokine may play a role in asthma [4], [5]. In humans, the IL-9 and *IL-9R* gene are located on chromosomal region where a linkage with asthma and its risk factors has been demonstrated[6], [7].Moreover, the development of transgenic mice over-expressing IL-9 has suggested a potential role for this cytokine in the development of airway eosinophilia, mast cell hyperplasia, mucus production and airway hyperresponsiveness[8], [9], [10]. Collectively, these findings support the concept that IL-9 may significantly be involved in mediating both pro-inflammatory and changes in airway responsiveness that characterizes the atopic asthmatic state.

The intracellular signalling induced by IL-9/IL-9R on transformed cell line has been characterized in details[11]. The binding of IL-9 to IL-9R induces the activation of JAK1 which promotes the phosphorylation of STAT1, 3 and 5[11], [12]. Furthermore, IL-9 can activate insulin receptor substrate -2 (IRS-2) pathway which subsequently induces the activation of PI3K[13].

Previously, we showed that human ASM express a functional IL-9R and activation through this pathway lead to CCL11 expression. We also documented IL-9R immunoreactivity in smooth muscle bundle of atopic asthmatics bronchial biopsies [14]. However the mechanism by which IL-9 mediates CCL11 expression in primary ASM cells is not fully understood. In this report, we demonstrated that IL-9 mediates CCL11 gene expression via a STAT-3 dependent pathway.

## Results

### IL-9 Induced CCL11 Gene Expression in ASM Cells Is Independent of STAT-6 Activation

**Figure 1:**
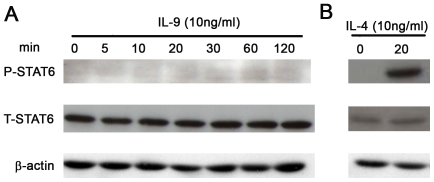
IL-9 does not induce STAT6 phosphorylation in human ASM cells. Growth arrested ASM cells were stimulated with IL-9 (A) or IL-4 (20 min,B) both at 10 ng/ml. Lysates were immunoblotted with phospho-specific Abs and detected by enhanced chemiluminescence as described in Methods. Total STAT6 and β actin Abs was used for loading control. The results represent one of similar results from four independent experiments.

We have previously demonstrated that IL-9 induces CCL11 expression in ASM cells[14]. Furthermore, CCL11 expression has been shown to be dependent on STAT-6 activation in various inflammatory and structural cells including ASM cells [15]. To determine STAT6 activation in response to IL-9 in ASM cells, total cell protein was probed with specific anti phospho-tyrosine STAT6 and total STAT6. As shown in Fig. 1 A, IL-9 stimulation did not induce STAT6 tyrosine phosphorylation over a 2 h time period in ASM cells. However, as expected IL-4 used as positive control induced a strong tyrosine phosphorylation of STAT6 (Fig. 1B).

**Figure 2:**
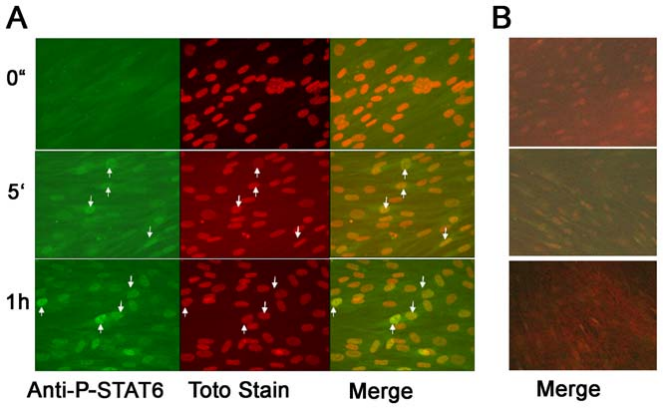
IL-9 does not induce STAT6 nuclei translocation in human ASM cells. Growth arrested semi-confluent ASM cells were stimulated with IL-4 (A) or IL-9 (B) both at 10 ng/ml in 8 wells slide. Slides were stained with specific anti- phospho tyrosine STAT-6 mAb or isotype matched control, followed by goat anti-mouse IgG F(ab')_2_ AlexaFluor® 488. Nuclear counter-staining was performed using TOTO-1 as described in Materials and Methods. Original magnification 400X.

Immunofluorescence coupled to confocal laser scanning microscopy was then preformed to determine STAT6 tyrosine-phosphorylation and translocation to the nucleus in IL-9 stimulated ASM cells. IL-4, used as a positive control, demonstrated noticeable STAT6 phosphorylation within 5 min in the cytoplasm and nuclei of ASM cells (Figure 2A). However, IL-9 did not induce noticeable STAT6 phosphorylation or translocation to the nuclei in ASM cells (Figure 2B). This data suggests that IL-9 induction of CCL-11 release may not be dependent on STAT6 activation.

### IL-9 Mediates CCL11 Expression in STAT6 Silenced ASM Cells

**Figure 3:**
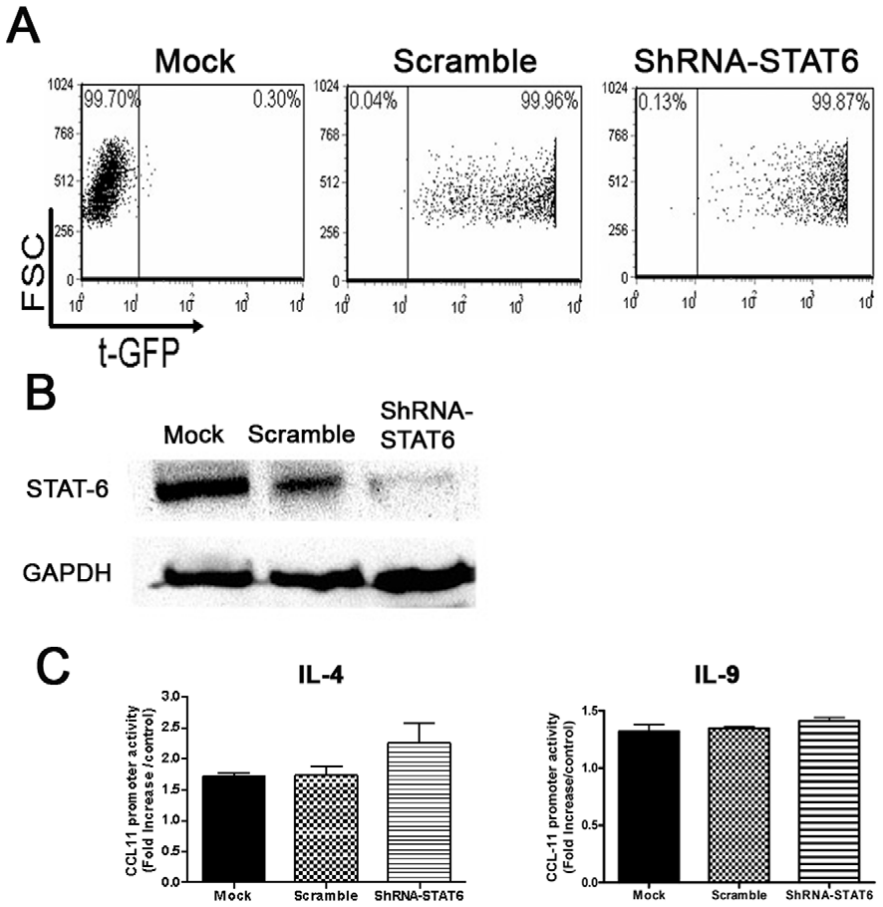
IL-9 mediated CCL11 expression is not affected in STAT6 silenced ASM cells. **A**. Human ASM cells were transduced by infecting with lentivirus containing scramble sequence, STAT6-shRNA sequence or mock and examined by flow cytometry for turbo GFP (tGFP) expression. **B**. Effect of STAT6-shRNA on STAT6 expression by ASM cells. Expression of total STAT6 in mock, scramble or STAT6-shRNA transduced ASM cells was analyzed by western blot. **C**. Stably silenced STAT6 ASM cells was co-transfected with CCL11 promoter then stimulated with IL-9 or IL-4 (both at 10 ng/ml) as described in Materials and Methods. The mean ±SE of three independent experiments are shown.

To confirm our previous data, we investigated whether the expression of CCL11 induced by IL-9 in ASM cells is affected by the absence of STAT6 using a genetic approach. Primary human ASM cells were transduced with a vector harboring STAT6-shRNA or scramble shRNA. FACS analysis showed more than 95% of shRNA transduced cells were tGFP+ (Fig. 3A) and resulted in a highly significant and reproducible decrease in STAT6 expression levels (Fig. 3B). Stably silenced STAT6 ASM cells were transfected with CCL11 promoter and stimulated with IL-9 (10 ng/ml), or positive control IL-4 (10 ng/ml)[16]. IL-4 or IL-9-induced transcriptional activation of CCL11 promoter was unchanged in STAT6 or scramble silenced ASM cells (n = 3, p<0.05, Fig. 3C).

### IL-9 Induces STAT3 Phosphorylation, Nuclei Translocation and Binding to CCL11 Promoter in ASM Cells

**Figure 4:**
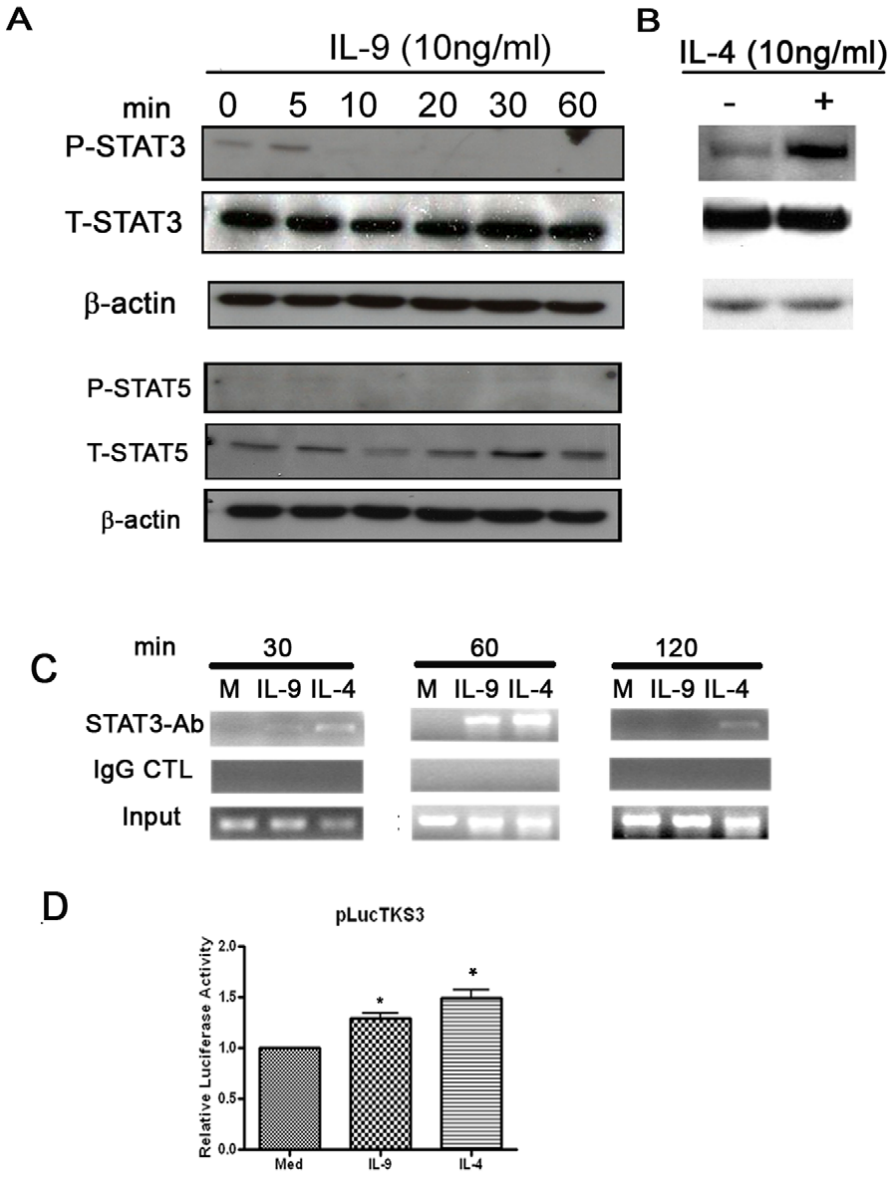
IL-9 induces STAT3 phosphorylation and *in vivo* binding to CCL11 promoter in ASM cells. A–B. Cells were stimulated with IL-9 (A) or IL-4 (only 20 min is shown in B) and analyzed as described in Figure 1. The data represent one of similar results from 5 independent experiments. C. IL-9 induced STAT3 binding to CCL11 promoter *in vivo*. Confluent and serum starved human ASM cells were treated with IL-4 (10 ng/ml) or IL-9 (10 ng/ml). The *in vivo* STAT3 binding to the CCL11 promoter was analyzed by ChIP assay as described under Material and Methods. The input represents PCR products from chromatin pellets prior to immunoprecipitation. The results are representative of three independent experiments with similar results. D. IL-9 driven STAT3 reporter gene activity. STAT3-specific reporter plasmid (p*Luc*TKS3) which harbors seven copies of a sequence corresponding to the STAT3-specific binding site was transfected into ASM cells or with pLucSRE serum response element (SRE) of the c-fos promoter (data not shown) following stimulation by IL-9 or IL-4 as described above. Data in D represent the mean ± SEM from a total of 5 independent experiments.

Previous studies have shown the involvement of both STAT5 and STAT3 in IL-9 signaling pathway in transformed cell lines [12], [17], [18]. To ascertain which STATs proteins were activated following ASM cells stimulation with IL-9, we performed western blot analysis using specific antibodies for the phosphorylated regulatory sites on STAT3 and STAT5. As shown in figure 4A, IL-9 induces rapid phosphorylation of STAT3 at Tyr705 within 5 min that diminishes afterward (Fig. 4A). No STAT5 phosphorylation could be detected upon IL-9 stimulation. IL-4, used as positive control, also induced a strong STAT3 phosphorylation (Fig. 4B) as described in B cells [19].

We then performed chromatin immunoprecipitation (ChIP) to investigate whether IL-9 stimulation induces STAT3 nuclei translocation and binding *in vivo* to CCL11 promoter. Equal amount of nuclear samples were immunoprecipitated with an antibody against STAT3, and the immunoprecipitated DNA was amplified with the specific human CCL11 primer pairs. The status of the STAT3 transcription complex at CCL11 promoter in response to IL-4 was used as positive control [16]. In contrast to unstimulated cells, IL-9 induced a marked enrichment of STAT3 associated CCL11 promoter DNA, 197 bp region between −136 to +61, at 30, 60 min which returned to baseline at 120 min (Fig. 4C). In contrast, immunoprecipitation with isotype control antibody resulted in the absence of a 197 bp band suggesting that STAT3 specifically binds to CCL11 promoter. Furthermore, IL-4 treatment resulted in a clear enrichment of STAT3 at 30, 1 and 2 h (Fig. 4C) as we previously described[16]. These data suggest that IL-9 induced CCL11 transcription involves *in vivo* STAT3 binding to CCL11 promoter.

We next used STAT3-specific reporter plasmid (p*Luc*TKS3) which harbors seven copies of a sequence corresponding to the STAT3-specific binding site in the C-reactive gene promoter [20] to confirm the activation of STAT3 mediated gene expression by IL-9. As showed in figure 4D, both IL-9 and IL-4 are able to drive STAT3 dependent luciferase promoter activity compared to p*Luc*TKS3 transfected unstimulated ASM cells (mean RLU ± SD for IL-9: 51897±4539; IL-4: 59316±9458; med: 40231±8766; P<0.05, n = 5). Furthermore, IL-9 and IL-4 are not able to induce luciferase promoter activity when cells are transiently transfected with pLucSRE serum response element (SRE) of the c-fos promoter (mean RLU ± SD for IL-9: 138±17; IL-4: 125±15; med: 98±23; n = 5, P>0.05).

### Cell-Permeable STAT3 Inhibitor Phospho-Peptide and STAT3 Dominant Negative Diminish IL-9 Mediated CCL-11 Promoter Activity

**Figure 5:**
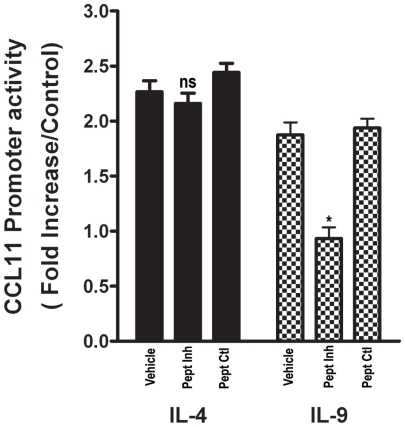
STAT3 inhibitory peptide decreases IL-9 mediated CCL11 transcriptional activity. Human primary ASM cells were growth-arrested, transfected with CCL11 promoter for 24 h. Cells were then incubated with inhibitory or control peptide for 1 h before stimulation for 12 hour with IL-4 or IL-9 after which transcriptional activation was measured by luciferase activity. Fold induction represents luciferase activity in cytokine treated cells compared to transfected untreated cells, and is the mean of five independent experiments. * P<0.05.

Primary ASM cells were first pre-treated with STAT3 phospho-peptide, a cell-permeable inhibitor that acts as a highly selective potent blocker of STAT3 activation[21], transfected with CCL11 proximal promoter followed by IL-9 stimulation. As a negative control, cells we pretreated with a STAT3 control peptide (STAT3-CP) that has the same residue sequence without phospho-group. As shown in figure 5, human ASM cells showed a significant reduction (p<0.05) in CCL11 luciferase activity in response to IL-9 stimulation following STAT3 inhibition with STAT3-IP but not with STAT3-CP. STAT3-IP did not affect IL-4 induced CCL11 promoter activity in ASM cells (Figure 5) as we described previously [16].

**Figure 6:**
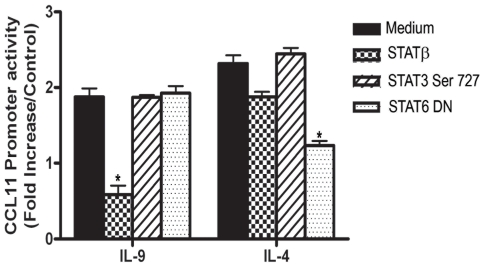
Effect of DN STAT3β, STAT3 Ser 727, or DN STAT6 over-expression on IL-9 induced CCL-11 promoter activity. Human primary ASM cells were co-transfected with WT-CCL-11 promoter and DN STAT3, STAT3β, STAT3 mutant at Ser 727 or DN STAT6. 24 h after transfection, cells were stimulated for 12 h with IL-4 or IL-9 (all at 10 ng/ml). Fold induction represents luciferase activity in cytokine treated cells compared to transfected untreated cells, and is the mean of five independent experiments. * P<0.05.

We then examined the effect of a dominant negative STAT-3 DN (STAT3β), STAT6 DN and STAT3 mutant at serine 727 on IL-9 mediated CCL-11 promoter activity in ASM cells. STAT3β is a naturally occurring splice variant with a deletion in the C terminal trans-activation domain that harbors the transcriptional activation domain and the Ser 727 residue [22], [23]. ASM cells co-transfected with CCL11 promoter construct and STAT3β expressing vector [20], but not with STAT3 mutant Ser 727, showed a significant decrease in luciferase activity in response to IL-9 stimulation (Fig. 6, n = 5, p<0.001). Interestingly, cells co-transfected with STAT3β expressing vector dispalyed a reduction in luciferase activity in response to IL-4 but was statistically significant (Fig. 6, n = 5, p<0.001). Furthermore, co-transfection with STAT-3 mutant at serine residu has no effect on IL-4 mediated CCL-11 promoter activity. This result suggests that serine phosphorylation is not involved in IL-9 or IL-4 mediated gene expression. Disruption of STAT6 function by co-transfection of STAT6 dominant negative vector significantly decreases IL-4 but not IL-9 induced CCL11 promoter activity (p<0.05, n = 5). These results confirm that STAT3 activation is involved in IL-9 induced CCL11 promoter activity.

### ShRNA Mediated STAT3 Silencing Abrogates IL-9 Mediated CCL11 Expression

**Figure 7:**
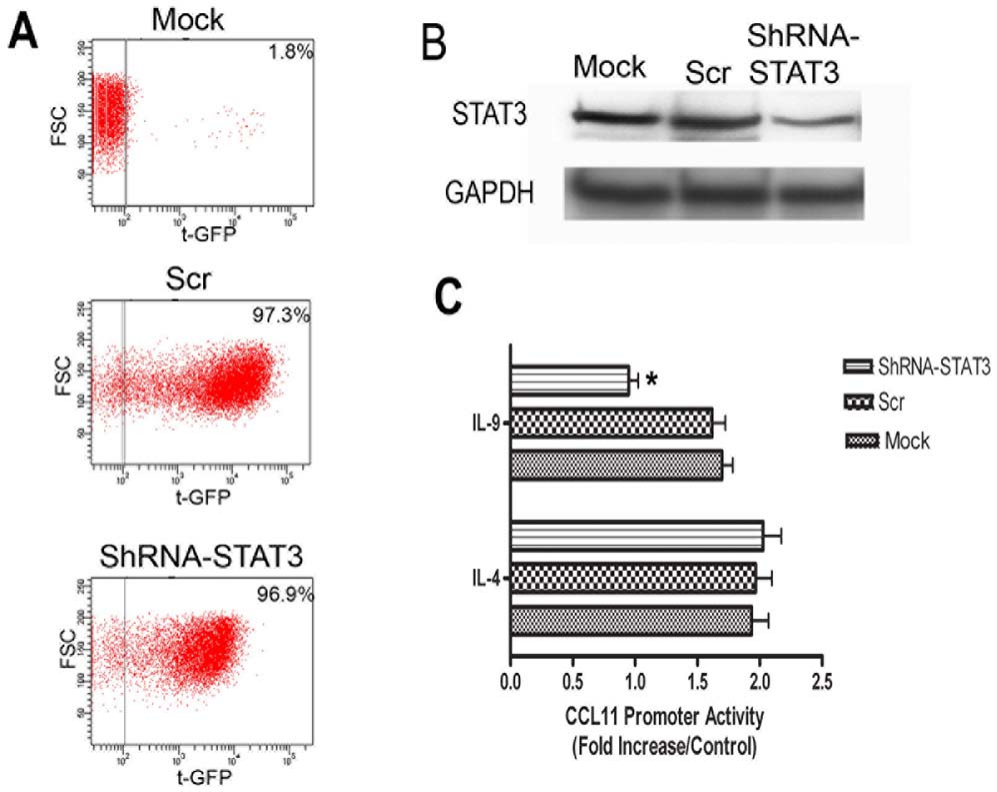
Silencing STAT3 abrogates IL-9 mediated CCL11 expression. **A**. Efficiency of Lentiviral transduction in human ASM cells. Human ASM cells were transduced by infecting with lentivirus containing scramble sequence or STAT3-shRNA sequence and examined by flow cytometry for tGFP expression. **B**. Total STAT3 in mock, scramble and STAT3-ShRNA transduced ASM cells as analyzed by Western blot **C**. ASM cells stably expressing scramble or STAT3-shRNA was transfected with CCL11 promoter luciferase reporter plasmid and stimulated with IL-9 (10 ng/ml) or IL-4 (10 ng/ml) as described in Methods. The mean ±SEM of three independent experiments are shown. * P<0.05 compared to scramble lentiviral transduced ASM cells stimulated with IL-9.

We then used shRNA to human STAT3 to inhibit STAT3 expression in ASM cells (Fig. 6A) [16]. Mock and scramble sequence was used as negative control. As revealed by FACS analysis, the transduction efficiency of ASM cells was more than 95% as showed by the expression of turbo green fluorescent protein reporter gene (tGFP+) (Fig. 7A). Moreover, STAT3 protein expression level was significantly reduced in STAT3-shRNA transduced ASM cells, effect that was not observed with scramble shRNA (Fig. 7B).

To determine if the transcriptional activation of CCL11 by IL-9 is affected in the absence of STAT3, stable STAT3 silenced ASM cells were transfected with CCL11 promoter and stimulated with IL-9 (10 ng/ml), or IL-4 (10 ng/ml). IL-9 induced transcriptional activation of CCL11 promoter was significantly reduced in STAT3-shRNA transduced cells but unchanged in scramble transduced cells (n = 5, p<0.05). Furthermore, CCL11 promoter activity induced by IL-4 was unaffected in STAT3 silenced ASM cells (Fig. 7C) [16]. To confirm the specificity of our shRNA strategy, STAT3 silenced ASM cells was also stimulated by IL-1β that is not dependent on the STAT3 pathway. CC11 promoter activity induced by IL-1β was similar in mock, scramble and STAT3 silenced ASM cells with a 3.2±0.5, 2.8±0.7 and 2.95±0.36 fold increase respectively compared to cells transfected with CCL11 promoter without IL-1β stimulation (data not shown).

## Discussion

Our previous work showed that primary ASM cells express a functional IL-9R *in vitro*; and *in vivo* ASM bundle from atopic asthmatics displayed higher expression of IL-9R compared to normal controls[14]. Activation of IL-9R in ASM cells mediated CCL11 expression that can enhance eosinophil chemotaxis[14]. In this study, we aimed to define the role of STAT3 in IL-9 mediated CCL11 expression in ASM cells. Using different strategies, we showed that IL-9 mediated CCL11 expression in ASM cells requires STAT3 but not STAT6 pathway. IL-9 induced rapid STAT3 activation in primary ASM cells that was not observed in case of STAT6 or STAT5. Furthermore, STAT3 binding to CCL11 promoter was also observed *in vivo* upon IL-9 stimulation of ASM cells. Disruption of STAT3 activity with SH2 domain binding inhibitory peptide results in significant reduction of IL-9 mediated CCL11 promoter activity. Naturally occurring DN STAT3β [22], [23] over-expression in ASM cells, but not Ser 727 STAT3 or STAT6 DN, abolishes IL-9 mediated CCL11 promoter activity. STAT3 but not STAT6 silenced ASM cells showed significant reduction in IL-9 mediated CCL11 promoter activity and mRNA expression. Our data strengthen the role of STAT3 as a major player in IL-9 mediated CCL11 expression in ASM cells.

CCL11 is a chemokine belonging to the CC family that has been shown to be a potent chemoattractant for eosinophils both *in vitro* and *in vivo* [24]. Increased production of CCL11 has been associated with allergic diseases such as asthma[25]. Th-2 cytokines, particularly IL-4 can induce CCL11 release in many structural cells including ASM cells [15], [26]. Previously, it has been demonstrated that human ASM cells express CCL11 following pro-inflammatory cytokine stimulation [25]. The CCL11 produced and secreted by ASM cells may then amplify the chemokine signal generated by infiltrating inflammatory cells in the airway, thereby augmenting the recruitment of eosinophils, basophils, and Th-2 lymphocytes to the airways[2]. The accumulation of these inflammatory cells may subsequently contribute to the development of airway hyperresponsiveness, local inflammation, and tissue injury through the release of granular enzymes and other cytokines. Our current study shows that IL-9 dependent activation of ASM cells can induce CCL11 via STAT3 pathway, thus revealing a new mechanism for induction of CCL11 within the airways.

STAT3 is a member of cytokine and growth factor inducible transcription factors. Upon phosphorylation at tyrosine residues, STAT3 undergoes conformational change, dimerizes and translocates to the nucleus where it can bind specific DNA motifs and activate the transcription of distinct groups of genes [27]. Studies using cell specific STAT3 KO indicates an important anti-inflammatory role of STAT3 in innate immunity[28]. Further evidences suggest a central role of STAT3 in regulating the anti-inflammatory response in skin and liver pathological models [28], [29]. However, targeted disruption of STAT3 in airway epithelial cells (e-STAT3-/-) showed a significant decrease of airway eosinophilia suggesting an important role for STAT3 in allergic inflammation [30]. Our present data extends these data by showing that STAT3 activation is critical for IL-9 mediated CCL11expression in ASM cells which may contribute to airway inflammation.

Upon activation, STAT3 protein can be phosphorylated at both Ser 727 and tyrosine 705 residues[27]. Our data showed that STAT3 Ser727 mutation over-expression in ASM cells has no effect on CCL11 gene expression. In transgenic mice expressing a form of the STAT3 Ser727 mutant [31], tyrosine phosphorylation of STAT3 was detected upon oncostatin M stimulation of fibroblast; and many of the downstream signaling pathways associated with STAT3 remain intact [31]. *In vitro*, similar results were observed in STAT3 Ser727 mutant transfected cells[22]. Taken together, these data are in agreement with our findings and suggest that STAT3 serine phosphorylation has no effect on transcription of CCL11 gene in ASM cells upon IL-9 stimulation. Besides STAT3, IL-9 has been shown to activate STAT1 and STAT5 in malignant cell line. In our data however, IL-9 failed to induce STAT1 (data no shown) or STAT5 phosphorylation. Furthermore, co-transfection of STAT5DN in ASM cells did not affect IL-9 mediated CCL11 promoter activity (data not shown).

In our study, IL-4 used as positive control induced STAT3 phosphorylation and recruitment to CCL11 promoter *in vivo*. However, STAT3 inhibition by using inhibitory peptide, DN STAT3β over-expression or shRNA targeting strategy failed to demonstrate any role of STAT3 in mediating IL-4-induced CCL11 promoter. This discrepancy can be explained by the fact that IL-4 can induce both STAT6 and STAT3 activation in ASM cells as has been demonstrated in other cells[19], [32] including ASM cells [16]. Indeed, we confirmed that IL-4 can induce STAT3 and STAT6 phosphorylation in ASM cells (Figure 1 and 4) and both can bind to the CCL11 promoter *in vivo* (Figure 4C). As such, IL-4 can mediate CCL11 expression using STAT6 or STAT3 dependent pathway in human ASM cells.

IL-9 has been shown to activate extracellular signal-regulated kinases (ERK) in ASM cells[33]. Furthermore, the MAPKs have been shown to play an important role in modulating STAT3 signaling. Specifically, ERK1/2 have been shown to phosphorylate STAT3 at Ser 727 and to play a crucial role for transcriptional activity [34], [35]. Data from our lab showed that pharmacological targeting of ERK1/2 inhibits IL-9 mediated CCL11 release and promoter activity (Data not shown). These results are in agreement with data by Baraldo *et al.* that demonstrated ERK1/2 activation by IL-9 in ASM cells[33] and suggest a possible cross talk between ERK and STAT3 pathway in ASM cells.

In summary, our study strengthens the role of STAT3 as a major player in IL-9 mediated CCL11 expression. Our results also highlight the importance of this pathway as a potential therapeutic target in airway allergic inflammation.

## Materials and Methods

### Reagents and Antibodies

Recombinant human IL-9, IL-4, IL-1β, human recombinant CCL11, anti-CCL11 capture mAbs and biotinylated detection mAbs are from R&D (Minneapolis, MN). Mouse IgG1 isotype control (clone MOPC21), and goat IgG were from Sigma (Oakville, Ontario). FITC conjugated rat anti-mouse IgG was from Jackson (West Grove, PA). Mouse IgG1 anti-phospho tyrosine specific STAT-5 and STAT-6 were purchased from BD (Mississauga, Ontario). Mouse mAb anti-phospho-tyrosine STAT-3 (Y705), affinity purified rabbit anti-total STAT-3, STAT-5 and STAT-6 were from Santa Cruz (Santa Fe, CA). Goat anti-mouse IgG (Fab)'_2_ Alexa 488, nuclear TOTO stain were obtained from Molecular Probes (Eugene, Oregon). Fetal bovine serum (FBS), RPMI 1640 and antibiotics (penicillin, streptomycin) were from Hyclone Laboratories (Logan, UT). Unless stated otherwise, all other reagents were obtained from Sigma Chemical Co (Oakville, Ontario).

### Isolation and Culture of Human Airway Smooth Muscle Cells

Human bronchial smooth muscle cells were obtained from macroscopically healthy segments of 2^nd^–4^th^ generation lobar or main bronchus of patients undergoing surgery for lung carcinoma in accordance with the procedures approved by the Ethics Committee of the University of Manitoba, Winnipeg, Canada. Informed consent for ASM harvesting was obtained from all patients. Primary airway smooth muscle (ASM) cells were isolated from explants as previously described[36]. Cells were cultured in Dulbecco's modified Eagle medium (DMEM) supplemented with 10% FBS, L-glutamine (2 mM), penicillin (100 U/ml), and streptomycin (100 µg/ml) at 37°C with 5% CO_2_. At confluence, primary human ASM cells exhibited spindle morphology and a hill-and-valley pattern that is characteristic of smooth muscle in culture. Moreover, ASM cells at confluence retain smooth muscle-specific actin, SM22, and calponin protein expression, and mobilize intracellular Ca^2+^ in response to acetylcholine, a physiologically relevant contractile agonist[36].

### Immunofluorescence and Confocal Laser Scanning Microscopy (CLSM)

Serum fed human ASM cells grown on 8 well glass slides (Naig Nunc, Napierville, IL) were cultured up 60–80% confluence. Cells were then stimulated with IL-4 or IL-9 both at 10 ng/ml. Slides were fixed with 4% paraformaldehyde, air-dried, and stored at −20°C until use. Slides were saturated with universal blocking solution for 10 min (DakoCytomation, Carpenteria, CA). Slides were incubated with 10 µg/ml of anti-phospho-tyrosine specific anti-STAT6 mAb or isotype matched control (MOPC21) in dilution buffer (Dako Cytomation) overnight at 4°C, washed twice with Tris-Buffered Saline (TBS) followed by incubation for 1 h at room temperature (RT) with goat anti-mouse IgG (Fab)'_2_ AlexaFluor® 488 (1:100 dilution) (Molecular Probe). Slides were then extensively washed with TBS and counterstained with nuclei stain TOTO for 2 min. After washing with TBS, the slides were then mounted with anti-fad agent (Molecular Probe). Samples were photographed on Olympus AX70 microscope with a Photometrics PXL cooled CCD Camera and Image-Pro® Plus Software (Carsen Group Inc, Ontario).

### Assessment of STAT3, STAT5, and STAT6 Phosphorylation

Nearly confluent ASM cells were growth arrested by FBS deprivation for 48 h as described above. Cells were then stimulated in fresh FBS free medium with IL-9 (10 ng/ml), IL-4 (10 ng/ml) or medium alone. At selected time points, the cells were washed once with cold phosphate buffered saline (PBS), and total proteins were extracted with lysis buffer (1% NP-40, PMSF, 2 mM sodium vanadate, 0.1% sodium deoxycolate, and protease inhibitor cocktail (Roche, Penzberg, Germany). Harvested lysates were centrifuged for 10 min at 4°C to pellet cellular debris. The supernatants were removed and stored at −70C. Protein lysate (10 µg) were loaded on 10% SDS PAGE, followed by transfer to nitrocellulose membranes (Invitrogen). The blots were then blocked with 5% non fat dry milk in TBS/0.1% Tween (TBST) for 1 h at room temperature, and then incubated overnight at 4°C with antibodies specific for phosphorylated STAT3 (Y705), STAT5 (Y694) and STAT6 (Y641). After washing with TBST, the blots were incubated with goat anti-mouse or goat anti rabbit horseradish peroxidase conjugated secondary antibodies and bands were revealed with ECL reagents (Amersham Pharmacia, Baie D'Urfe, Quebec, Canada). After stripping, total anti- STAT3, STAT5, STAT6 and β actin were used as loading control.

### Luciferase Reporter Constructs and Cell Transfection

Plasmids expressing a dominant negative (DN) form of STAT3 (pSG5hSTAT3β) and a STAT3 mutant at serine 727 (S727A) were kindly donated by Dr. R. Fostra (Royal Cancer Hospital, Brampton UK) and Dr. R. Jove, (H. Lee Moffitt Cancer Center and Research Institute, Tampa, Florida) respectively. A Stat3-specific reporter plasmid (p*Luc*TKS3) which harbors seven copies of a sequence corresponding to the STAT3-specific binding site in the C-reactive gene promoter (termed APRE, TTCCCGAA) and pLucSRE, which contains two copies of the serum response element (SRE) of the c-fos promoter both upstream from a firefly luciferase coding sequence were donated by Dr. J. Turkson[36] (University of Central Florida College of Medicine, Orlando, FL). STAT6 DN and eotaxin-1/CCL11-WT promoter luciferase construct were provided by J. Hoeck (Salzburg University, Austria)[14]. ASM cells (4×10^4^) were seeded into 12-well culture plates in fresh complete DMEM. After 24 h at 50–70% confluency, cells were transfected transiently using ExGen 500 *in vitro* transfection reagent (Fermentas, Ontario, Canada) according to the manufacturer's instructions. In each well, 1.6 µg of CCL11 promoter-luciferase DNA and 0.4 µg of *Renilla* luciferase reporter vector-pRL-TK (Promega) were co-transfected for 24 h. In some experiments, DN STAT3, STAT6 or STAT3 Ser 727 mutant were co-transfected with WT CCL11 promoter-luciferase DNA. The medium was changed and cells were washed and stimulated with IL-9 or IL-4 (both at 10 ng/ml). After 12 h of cytokine stimulation, cells were washed twice with PBS and cell lysates were collected with 100 µl of reporter lysis buffer (Promega). The luciferase activity was measured by the Dual-Luciferase Assay System kit (Promega) using a luminometer (model LB9501; Berthold Lumat). Briefly, 20 µl of cell lysate was mixed with 100 µl of Luciferase Assay Reagent II (Promega) and firefly luciferase activity was first recorded. Then, 100 µl of Stop-and-Glo Reagent (Promega) was added, and *Renilla* luciferase activity was measured. All values are normalized to *Renilla* luciferase activity and expressed relative to the control transfected non stimulated cells.

### Chromatin Immunoprecipitation (ChIP) Assay

Chromatin immunoprecipitation assay was done essentially as described elsewhere[37], with minor modifications. Briefly, human ASM cells in 90-mm dishes were grown to confluency and serum starved for 48 h. Cells were then incubated with or without IL-9 or IL-4 (both at 10 ng/ml) for 30, 60 or 120 min. The cells were cross linked for 10 min with 1% formaldehyde in PBS to fix protein-DNA complexes, washed and lysed in 200 µl of SDS-lysis buffer (50 mM Tris, pH 8.0; 1% SDS; 5 mM EDTA; complete proteinase inhibitor cocktail) and sonicated on ice. The samples were then centrifuged for 10 minutes, and chromatin pellets were sheared by sonication. Samples were pre-cleared with salmon sperm DNA-saturated protein A agarose (Upstate Biotech, Waltham, MA) for 1 h at 4°C with rotation. After centrifugation at 500×*g* for 1 min, one portion of the soluble chromatin was used as DNA input control, and the remains were sub-aliquoted and then precipitated using specific antibodies, a non-immune rabbit immunoglobulin G (IgG; Santa Cruz) or STAT3 rabbit mAb separately, overnight at 4°C. Protein-bound immunoprecipitated DNA was washed successively with low-salt buffer (0.1% SDS, 1% Triton X-100, 2 mM EDTA, 20 mM Tris-HCl [pH 8.1], 150 mM NaCl), high-salt buffer (same as the low-salt buffer but with 500 mM NaCl), LiCl buffer (0.25 M LiCl, 1% NP-40, 1% deoxycholate, 1 mM EDTA, 10 mM Tris-HCl [pH 8.1]), and Tris-EDTA (pH 8.0) and then eluted with elution buffer (1% SDS, 100 mM NaHCO_3_). The elution (cross-linking of protein-DNA complexes) was reversed by incubation with 5 M NaCl at 65°C for 4 h, and then incubated for 1 h at 45°C with 10 mg of proteinase K (Sigma) per ml. DNA was then extracted with phenol-chloroform, precipitated with ethanol, and resuspended in 20 µl of TE buffer. The DNA from both sample and input was amplified by semi-quantitative PCR (30 cycles) using the primers specific for the CCL11 promoter (–136 to +61) encompassing both STAT and NF-κB binding sites. Primer pairs of CCL11 promoter are: forward, 5′-CTTCATGTTGGAGGCTGAAG-3′, and reverse, 5′-GGATCTGGAATCTGGTCAGC-3′. PCR products were resolved by using 3% agarose gel and visualized with ethidium bromide.

### Inhibitory Peptide

A cell-permeable STAT3 peptide inhibitor that consist of H-PY*LKTK-AAVLLPVLLAAP-OH, and control peptide 2) H-PYLKTK-AAVLLPVLLAAP-OH, were described previously[21]. The control peptide is identical in sequence to the inhibitor except that the tyrosine residue, which is essential for the inhibitory action, is not phosphorylated[21]. The peptides were synthesized by the Bio S&T Company (Montreal, Quebec, Canada). Peptides were used at concentrations of 500 µM[21].

### Lentiviral Vector Transduction in Human ASM Cells

293T cells used for virus production and titration were cultured in Iscove's modified Dulbecco's medium (HyClone, Logan, UT) supplemented with 10% fetal bovine serum (FBS), and 1% penicillin/streptomycin/glutamate (PSG) (Gibco, Grand Island, NY). Lentiviruse were generated and titrated using 293T cell lines as previously described [38]. For gene silencing, a clone specific for STAT3 (clones Id: V2LHS_262105, V2LHS_88502) was obtained from Open-Biosystems (Huntsville, AL). A control scrambled shRNA unrelated to STAT3 or STAT6 sequences was used as a negative control for lentiviral transduction. For all gene silencing studies, ASM cells were transduced at 10 infectious unit per ml in the presence of polybrene (8 µg/ml). In brief, cells were exposed to recombinant lentivirus for 2 hr at 37° C, medium replaced and cultured for additional 72 hrs. Transduced cells were selected with puromycin (4 µg/ml).

### Statistical Analysis

Data were obtained from experiments performed in triplicates and repeated at least three times, and results are expressed as geometric mean ± SD. Statistical significance was determined using a *Mann-Whitney U* test and P values <0.05 were considered statistically significant.
